# Lung Dendritic Cell Developmental Programming, Environmental Stimuli, and Asthma in Early Periods of Life

**DOI:** 10.1155/2012/176468

**Published:** 2012-11-07

**Authors:** Shanjana Awasthi, Bhupinder Singh, Robert C. Welliver, Rodney R. Dietert

**Affiliations:** ^1^Department of Pharmaceutical Sciences, College of Pharmacy, University of Oklahoma Health Sciences Center, Oklahoma City, OK 73117, USA; ^2^Department of Pediatrics, University of Oklahoma Health Sciences Center, Oklahoma City, OK 73104, USA; ^3^Department of Microbiology and Immunology, Cornell University, Ithaca, NY 14853, USA

## Abstract

Dendritic cells (DCs) are important cells of our innate immune system. Their role is critical in inducing adaptive immunity, tolerance, or allergic response in peripheral organs—lung and skin. The lung DCs are not developed prenatally before birth. The DCs develop after birth presumably during the first year of life; exposures to any foreign antigen or infectious organisms during this period can significantly affect DC developmental programming and generation of distinct DC phenotypes and functions. These changes can have both short-term and long-term health effects which may be very relevant in childhood asthma and predisposition for a persistent response in adulthood. An understanding of DC development at molecular and cellular levels can help in protecting neonates and infants against problematic environmental exposures and developmental immunotoxicity. This knowledge can eventually help in designing novel pharmacological modulators to skew the DC characteristics and immune responses to benefit the host across a lifetime.

## 1. Introduction

Asthma is a serious pulmonary disease that affects about 300 million people worldwide [1], and 8.2% (about 25 million) of the population within the USA [2]. A significant number of patients develop asthma during early childhood. A number of cross-sectional and longitudinal cohort studies in adult asthmatic patients suggest that the childhood asthma poses a risk for more severe asthma or relapse during adulthood [3–6]. One among ten children has asthma, and this trend has increased over the recent years [7]. Characteristics (airway obstruction, airway hyperresponsiveness, atopy, and recent wheeze) observed in children have been reported as predictors of asthma symptoms in adulthood. This is supported by evidence that the sensitization to allergens at young age increases the likelihood of asthma in adulthood [8–11]. 

**Figure 1:**
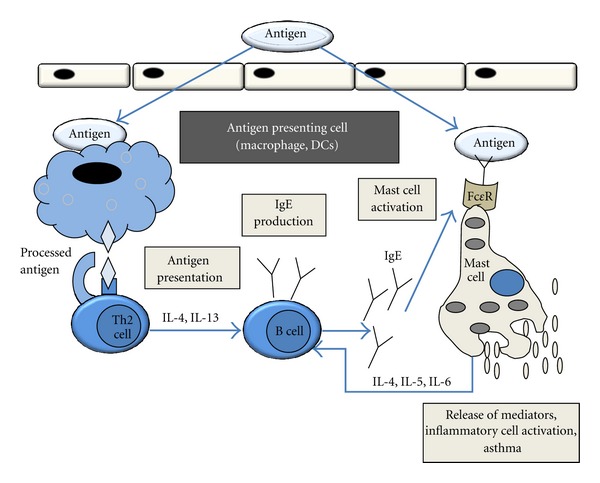
An illustration depicting the types of immune cells involved in a Th2-mediated allergic response. Antigen presenting cells (macrophages or DCs) take up the antigen, process it, and present it on the MHC molecule on the cell surface. The antigen presenting cells induce naïve T cells towards Th1 or Th2. Th2 response is mainly responsible for downstream events that include activation of B cells, production of IgE, and binding of IgE to Fc*ε* receptor on the cell surface of mast cells, resulting into mast cell degranulation and inflammation.

In allergic asthma, an immune reaction is caused by inhaled allergens with an overwhelming inflammatory response and obstruction in the airways. As a first step in sensitization, the antigen presenting cells (dendritic cells—DCs, macrophages, and lung epithelial cells) take up and process the inhalant allergens. The DCs are recognized as the key immune sentinel cell in the peripheral organs, including lung [12], and are at the cross-roads of inducing tolerance or inflammation [13]. The DCs are activated directly or via cell-cell interaction [14]. The activated DCs in turn stimulate T and B cells and other immune cells, which release a variety of cytokines, chemokines, and chemical mediators. These mediators are responsible for affecting the local microenvironment and generating inflammation and obstruction in airways. Traditionally, asthma has been known as the Th2-mediated disease (Figure 1). Both Th2- and non-Th2-dependent immune elements and mechanisms are now recognized for a number of phenotypes and endotypes of asthma [15–17]. 

While asthma phenotypes and endotypes are not fully characterized, resident lung DC types could be important [12, 18–22]. Lung DCs exhibit unique phenotypes than those present in other organs or in circulation [23], and are distributed throughout the alveolar epithelium, alveolar parenchyma, and nasal mucosa [24, 25]. A variety of investigations in rodents and human patients have reported the importance of different lung DC types in asthma [26–29]. This corresponds with the reported results on alterations in selective DC populations in the bronchoalveolar lavage fluids (BALFs) and in peripheral blood of patients with asthma (Table 1). A number of distinctive reports are available in the literature on the characteristics and functions of lung DC types [23, 30, 31], and are not reviewed here. In this paper, we provide an overview of lung DC development, exposure to pathogens, allergens, and environmental chemicals during early childhood, and their long-term impact on asthma development. 

## 2. Critical Window of Immune Vulnerability

**Figure 2:**
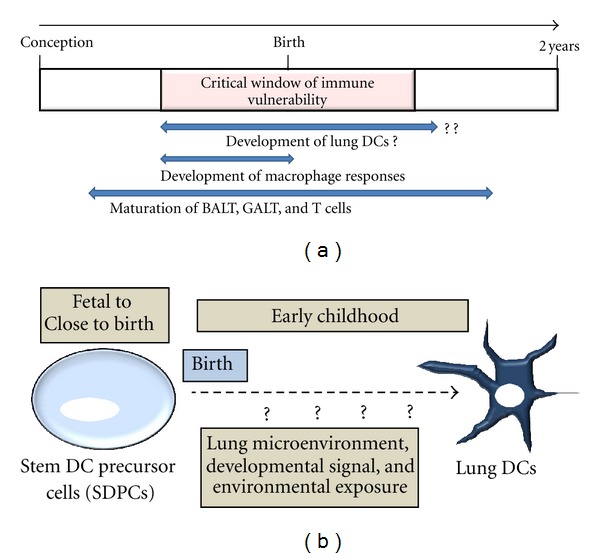
Immune development during critical window of vulnerability. (a) Timeline of maturation of bronchus-associated lymphoid tissue (BALT), gut-associated lymphoid tissue (GALT), T cells, and macrophages. (b) Lung-resident SDPCs could be the plausible source of lung DCs in early childhood. The timing of lung DC development, environmental factors triggering this transition, and signaling mechanisms involved in DC development remain unknown.

Over the last several years, prominent research studies have demonstrated that the late fetal and early postnatal periods are phases of reduced immune competence [12, 32, 33]. This reported reduced immune competence corresponds with the immaturity of immune system. In particular, the lung DCs are underdeveloped prenatally and close to term at birth [18, 34–38]. The development of lung DCs during infanthood has not been studied. Concurrent exposures to allergens and other environmental factors during this “critical window of immune vulnerability” have the potential to program DC types, DC functions, and DC-mediated tolerance or sensitization, which can have long-term respiratory and immunological consequences (Figure 2) [32, 39, 40]. 

## 3. Developing Immune System and Exposure to Asthma-Triggering Agents in Early Childhood

Particular events during early childhood can set the stage for specific developmental programming of DCs. The flexibility of a developing immune system and simultaneous exposure to allergens and other environmental stimuli can be important compounding factors for both the establishment and a long-term persistence of asthma. 

In addition to allergens, other risk factors for DC developmental programming towards particular DC phenotype and function during this “critical window period” may include environmental chemicals, drugs, certain dietary factors, infectious agents, and physical and psychological stressors. Not surprisingly, research studies suggest that the maturation and function of DCs are shifted by some of these predisposing risk factors. These include heavy metals, such as lead [41], and air pollutants particularly those from traffic [42] and environmental tobacco smoke [43]. Among relevant shifts that have been reported are: (1) reduced expression of Toll-like receptors (TLR)2 and TLR4 [44], (2) shift to Th2-biased adaptive immune response [45], and (3) promotion of misregulated (unresolved) inflammation [46, 47]. 

A number of recent studies have demonstrated that early exposures to *Chlamydia muridarum* (an intracellular pathogen) [48–50], Bacillus Calmette-Guérin (BCG) [51], and influenza A virus [52] alter the immune responses against allergens in adulthood via affecting the DC types and functions. Bacterial infections or stimulation with TLR4 ligand (Gram-negative bacteria-derived lipopolysaccharide) have been shown to skew the T-cell response to Th1 type during childhood [53]. In this regard, “Probiotics” and “Hygiene hypothesis” have been discussed elsewhere in the literature for controlling asthma-related immune response [54, 55]. 

## 4. Respiratory Syncytial Virus (RSV) Infection and Asthma

Respiratory syncytial virus infection is the most common cause of bronchiolitis and pneumonia in children under 1 year of age (Centers for Disease Control and Prevention, Atlanta, GA, USA). Severe forms of the RSV lower respiratory tract infections (LRTI) are characterized by airway obstruction and prominent wheezing. Furthermore, RSV infection in infancy has been linked to the development of asthma in childhood. Thus, there has been great interest in determining whether the pathogenesis of RSV bronchiolitis in infancy induces a persistent Th2 bias, leading to the development of Th2-dependent asthma in later childhood.

Some studies have focused on the expression of the prototype Th1 cytokine, interferon gamma (IFN-*γ*), and the Th2 cytokine, interleukin-4 (IL-4). Bendelja and colleagues studied the expression of IFN-*γ* and IL-4 in peripheral blood lymphocytes (PBL) of infants with various forms of RSV infection [56]. Among RSV-infected infants, the percentage of PBL positive for IL-4 was slightly greater than the percentage positive for IFN-*γ*, thus suggesting a Th2 bias. However, the expression of IL-4 was greater in subjects with mild upper respiratory tract infection (URTI) than in subjects with bronchiolitis or pneumonia. IFN-*γ* expression remained unaffected. Therefore, patterns of IFN-*γ* and IL-4 expression by PBL could not be associated with the severity of RSV infection. 

Others have determined the quantities of IFN-*γ* and IL-4 cytokines in respiratory tract secretions of infants with RSV infections. In one study, IFN-*γ* was found to be the predominant cytokine in subjects with all forms of respiratory tract illness related to RSV infection, with slightly higher ratios of IFN-*γ* to IL-4 in those with LRTI in comparison to those with URTI alone [57]. A second, larger study similarly demonstrated that the Th2 cytokines, IL-4, IL-5, and IL-13, were usually undetectable in secretions from infants with all forms of RSV infection. IFN-*γ* appeared to be protective against severe illness, in that IFN-*γ* concentrations were greater in subjects with milder, nonhypoxic forms of RSV-induced LRTI than in those with more severe LRTI accompanied by hypoxia [58]. In all of these studies, the differences in ratios of IFN-*γ* to Th2 cytokines were determined only by variations in IFN-*γ* concentrations between the groups. The findings of subsequent studies have also suggested a protective role for IFN-*γ* in RSV infection of infants [59, 60]. 

How the DC phenotypes and DC-induced T-cell responses are skewed following RSV infection is an important question, and could provide clues to the severity of the disease and predisposition to asthma. Infection of human infants with RSV and other viruses is followed by the appearance of DCs in nasopharyngeal and tracheal secretions [61, 62]. RSV infection of monocyte-derived DCs causes maturation of the cells, with expression of costimulatory molecules that participate in the instruction of T cells. However, it results in impaired CD4-positive T cells [63]. Although DC types were not addressed, the lung tissues from infants with fatal RSV demonstrated a lack of CD8−positive T cells [64]. Studies in mice have suggested unique roles for myeloid (mDC) and plasmacytoid DCs (pDC) in RSV infection. The pDCs in RSV-infected mice reduce the viral replication, while depletion of pDCs results in enhanced inflammatory responses and greater airway hyperreactivity [65]. A balance between mDC and pDC seems to determine the immune responses to RSV and airway reactivity following RSV infection [66]. The recruitment and activity of DC subsets occurring after RSV infection could skew immune responses toward either Th1 or Th2 cytokine pathways, thereby determining the eventual development of atopic disease or long-term airway hyperreactivity following RSV infection in infancy. 

## 5. Lung DC Development 

Despite growing understanding about the DC characteristics and functions in adult patients and animal models, the natural processes of lung DC development in prenatal or neonatal phase, as well as differentiation, maturation, and functional specialization of DCs, have not yet been studied. An understanding of perinatal and infant DC maturation in environmentally exposed tissues (including lung) is critical to better manage immune maturation for a healthier life course.

Histological details reveal that the MHC class II-positive cells start to appear in lung tissues of rat and human fetuses at 30–58% of term. Since the MHC class II is expressed ubiquitously by a variety of immune and nonimmune cells, the interpretation may not be DC specific. The appearance of MHC class II-positive DCs increases only after birth [37, 67–70]. 

Importantly, the airway structures and epithelial cell system are also not fully developed at the time of birth or during the neonatal period. It is reasonable to believe that direct cell-cell interaction or chemical mediators and growth factors released by another cell type can have a significant impact on the establishment of normal lung DC infrastructure as the lung microenvironment and the normal lung physiology evolve [13, 71, 72]. Repeated exposure to potentially harmful allergens or other environmental stimuli during this period can significantly affect the DC programming, phenotypes, and functions. Although it remains to be studied, these events may prompt a long-term memory for the generation of asthma-promoting DCs.

## 6. Animal Models for Studying Lung DC Development

Technical and ethical issues related to the availability of neonatal and infant lung tissues limit the enthusiasm to conduct studies that address the issues related to lung DC development. Studies are limited to first challenging the neonatal animal with infectious or allergenic stimuli and then investigating the DC types and functions later in life in the same animal. This approach may not adequately reflect the dynamic process of DC development or programming during early childhood in humans. Since DCs make <1% of total lung cells [34], it is not possible to harvest sufficient DC populations from rodent pups because of their small-sized lung. Hundreds of small-sized, age-matched mouse pups (most commonly used model) would be needed to harvest an adequate number of cells; it is almost impossible to have simultaneous births and enough age-matched mouse progeny available for this purpose. Also, the cells cannot be pooled from pups born close together, because murine pups (between birth and one-month) age at 150 times faster rate than humans [73]. There are significant differences in the lung anatomical [74], developmental, and immunological aspects of humans and mice (e.g., the lymphocyte and neutrophil distribution in blood, DC phenotypes) which make it difficult to interpret and translate the results to human infants [75–77]. As such, murine DC precursors/DC phenotypes are different from those reported in humans [23, 78]. Cellular intermediates within the hematopoietic stem cell hierarchy tree have been identified in tissues of mice and humans [79, 80]; significant differences have been noted in regards to their subsets and frequency [78]. Lack of reagents and paucity of information on DC-precursors or DCs limit such studies in other rodents. These studies are also not possible in human neonates or infants due to ethical reasons. Large animal models are expensive and require diligent work; these models can mimic the conditions of human infants very closely. To this effect, an asthma model is available in *Rhesus* monkeys. Similarities have been reported in asthmatic response among *Rhesus *monkeys and humans; the house dust mite antigen induces asthma conditions with clinical profiles; the biochemical and immunological markers are similar to those in human patients of asthma [74, 81, 82]. 

The baboon model seems ideal for studying the early human immune maturation and the developmental programming [83] because of similarities in ontogeny, immunology, reproductive physiology, placentation, and maternal-fetal transfer [84–88]. They are very close to humans in the evolutionary tree [89], and the lung development pattern in preterm baboons is similar to that found in preterm human babies [90]. Some of the immunological aspects [91–93], the bronchoconstriction, and the airway response against platelet-activating factor [94] in baboons are also analogous to those of humans and asthmatic patients, respectively. The advantages of the baboon over other commonly used primates, such as *Rhesus *monkey, include the ease of timed pregnancies due to the estrogen-sensitive sex skin in cycling females, the availability (baboons breed year-round), and the relative ease of handling (reviewed in [91]). Moreover, it allows harvesting of sufficient number of cells of interest from relatively large tissues of neonate and infant baboons.

**Figure 3:**
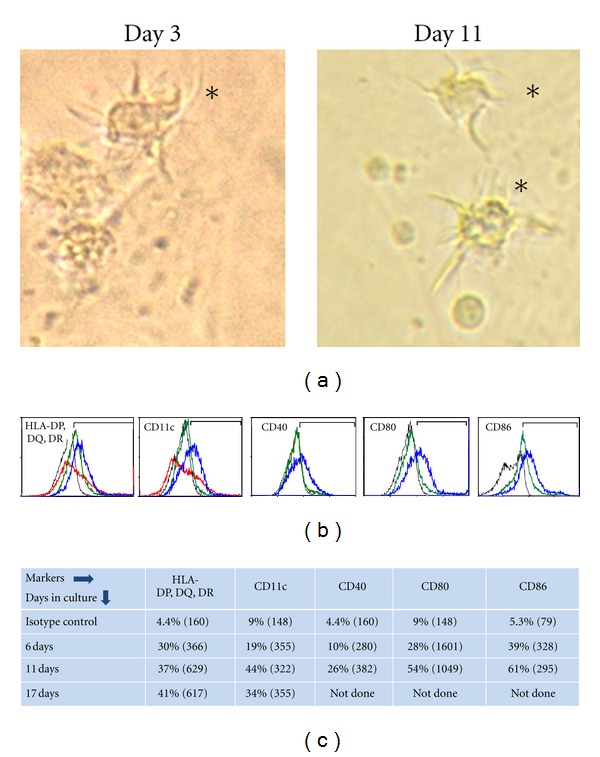
The SDPCs harvested from a close-to-term fetal baboon differentiate into DCs when cultured in presence of GM-CSF, IL-4, and TNF-*α*. The lung SDPCs were harvested on OptiPrep density gradient as per the method published earlier [34]. (a) Photomicrograph showing cells with dendrites (*). (b) Flow cytometry data showing increase in DC-marker expression. Black line: isotype control antibody-stained cells, green line: 6 days, blue line: 11 days, red line: 17 days—cells stained with antibodies to particular marker. (c) Data in the table shows % cells (fluorescent intensity) gated in the marked region of histogram charts in (b) staining positive for the specific marker.

We have studied the development of pulmonary innate immunity, including DCs, in a non human primate baboon (*Papio* species) model [34, 35, 95–97]. We have investigated the DC phenotypes and functions in prematurely delivered and close-to-term baboons (67–95% of gestation). Our results demonstrate that lung DC population having low density (similar to those of adult baboon lung DC population) remains underdeveloped until close to birth [34]. Since we do not know the stage of differentiation and the cell subsets, it is probably more appropriate to call them stem DC precursor cells (SDPCs) [36]. We have recently observed that the SDPCs can differentiate into DCs *in vitro* under DC-promoting conditions (Figure 3). A significant increase in expression of DC markers and tentacles is observed over time. It has been proposed by others that the tissue DCs can be generated from hitherto unknown resident stem cell populations. Isolation of stem cells has been reported from adult human lung [98]; their differentiation into lung DC-precursors or DCs has not yet been studied. 

## 7. Molecular and Immunologic Basis for DC Programming and Therapeutic Opportunities

In summary, our results indicate that the lung DCs are not developed at least until birth. We do not know when the DCs develop after birth during childhood, what triggers the lung DC development, and how the pathogenic stimuli affect the DC development leading to DC phenotypes with different functions. We speculate that pathogenic stimuli, such as allergens, may alter the lung DC developmental programming or SDPC→DC transition during early childhood in a way that an immunological memory is created for generation of asthma-promoting DC phenotypes or immune responses. Persistent reexposure to allergens may bolster the generation of these DC phenotypes so that an allergic response is maintained for a long time. 

An understanding of the basic immunobiology of DC development and the early programming of asthma-promoting DC phenotypes and functions against allergenic stimuli can pave the way to identify the basis of childhood asthma. Although it can be challenging to study the molecular mechanisms for lung DC development *in vivo*, studies with baboon lung SDPCs and DCs can provide useful tools to unravel the molecular and immunologic basis of the lung DC development and develop novel pharmacological modulators. A focused effort in this direction can provide a unique opportunity to effectively manage the pediatric immune system for optimized maturation and reduced health risks. This would include opportunities to intervene at an early age and skew the DC programming towards normal DC phenotype and function using pharmacological modulators. Additionally, this information may be useful in better protecting neonates and infants from environmental insults that increase the later-life health risks. It is the magnitude and persistence of downstream immunoinflammatory effects of tissue DC function that position this topic as a central health issue for allergic and other chronic diseases.

## Figures and Tables

**Table 1:** DC subsets in patients with asthma.

Clinical Condition	Altered DC phenotypes	References
Asthma	pDC (HLA-DR+, CD123+) increased in BALF mDC (HLA-DR+, CD11c+) increased in BALF	[99]
Allergic asthma	mDC (BDCA-3+, mannose receptor+) increased in BALF	[100]
Allergic asthma	pDC (BDCA4+) with increased Fc*ε*RI in blood mDC (CD1c+) with increased Fc*ε*RI in blood	[101, 102]
Asthma	Increased DC proportions in peripheral blood	[103]
Allergic asthma patients challenged with allergen	Increased pDC and mDC in sputum	[104]
Asthma	Increase in pDC1 and pDC2 expressing Fc*ε*RI	[105]
Repeated exposure to allergen	Depletion of mDCs	[23]
Asthmatic children	DC2 (CD11c−, CD123high+) decreased in blood	[106]
Asthmatic patients	Increased CD1a+ cells in bronchial mucosa	[107]
Experimentally elicited allergic rhinitis	pDC increased in nasal mucosa	[108]
Asthma	pDC increased; decreased mDC : pDC ratio in blood	[109]
Children with asthma	Deficiency of circulating pDC	[110]
Atopic patients with chronic rhinosinusitis	Increased Fc*ε*RI on DC (CD1+)	[111]

Abbreviations: BALF: bronchoalveolar lavage fluid, mDC: myeloid DC, pDC: plasmacytoid DC, BDCA: blood dendritic cell antigen.
